# Protective Factors for Early Psychotic Phenomena Among Children of Mothers With Psychosis

**DOI:** 10.3389/fpsyt.2018.00750

**Published:** 2019-01-14

**Authors:** Simon Riches, Louise Arseneault, Raha Bagher-Niakan, Manar Alsultan, Eloise Crush, Helen L. Fisher

**Affiliations:** ^1^King's College London, Social, Genetic & Developmental Psychiatry Centre, Institute of Psychiatry, Psychology & Neuroscience, London, United Kingdom; ^2^South London & Maudsley NHS Foundation Trust, London, United Kingdom; ^3^King's College London, Department of Psychosis Studies, Institute of Psychiatry, Psychology & Neuroscience, London, United Kingdom

**Keywords:** child and adolescent mental health, early intervention, familial psychosis, maternal psychosis, prevention, protective factors, psychotic experiences, resilience

## Abstract

**Background:** Early identification of sub-clinical psychotic experiences in at-risk individuals is vital to prevent the development of psychosis, even before prodromal symptoms emerge. A widely-replicated risk factor is having a family member with psychosis. The Environmental Risk (E-Risk) Longitudinal Twin Study has shown that better cognitive functioning, a stimulating family environment, and a cohesive community, are protective against psychotic experiences among children; while engaging in physical activity, social support, and a cohesive community are protective for adolescents. In the current study we investigate whether these factors also protect against the development of sub-clinical psychotic phenomena among children and adolescents in this cohort who are at high-risk of psychosis by having a mother with psychosis.

**Methods:** Data were utilized from the E-Risk Longitudinal Twin Study, a nationally-representative cohort of 2,232 twin children born in England and Wales in 1994–1995 followed to age 18. Psychotic phenomena were assessed in private interviews with children at ages 12 and 18, and mothers were interviewed about their own experiences of psychosis when children were aged 10 and 12. Bivariate and multivariate logistic regression analyses explored associations between individual, family, and community-level putative protective factors and absence of age-12 psychotic symptoms and age-18 psychotic experiences in children whose mothers had a diagnosis of a psychosis-spectrum disorder and/or reported psychotic symptoms.

**Results:** Higher IQ (OR = 0.97, 95% CI 0.94–1.00, *P* = 0.036) and living in a more socially cohesive neighborhood (OR = 0.88, 95% CI 0.79–0.98, *P* = 0.023) were independently protective against age-12 psychotic symptoms among children of mothers with psychosis. Higher levels of perceived social support were independently protective against age-18 psychotic experiences among children of mothers with psychosis (OR = 0.92, 95% CI 0.87–0.98, *P* = 0.006). However, there were no significant interactions between these protective factors and maternal psychosis in relation to an absence of childhood or adolescent psychotic phenomena in the full sample, indicating that protective effects were not specific to this group of high-risk children.

**Conclusions:** These findings provide preliminary evidence that preventive interventions for early psychotic phenomena could focus on improving cognition, social support, and cohesiveness of the local community. Given scarce resources these might usefully be targeted at high-risk children.

## Introduction

Psychotic disorders, especially schizophrenia, are responsible for a substantial proportion of disability worldwide (1), are associated with 10–25 years shorter life expectancy (2), and place a huge burden on families, health services, and society (3–5) Current treatments are unable to provide a cure; therefore, early identification of at-risk individuals may help to prevent the development of psychosis. Interventions in the prodromal stage have yielded some success but often fail to prevent poorer functional outcomes in the longer-term (6). It may therefore be necessary to intervene even earlier, before prodromal symptoms have emerged (7).

One potential target for preventative interventions could be sub-clinical psychotic experiences. These experiences, such as hearing voices, having visions, or feeling extremely paranoid, are relatively common in the general population, especially among young people. A systematic review and meta-analysis of population-based studies concluded that the median prevalence of psychotic symptoms was 17% among children aged 9–12 years, and 7.5% among adolescents aged 13–18 years (8). Early-life psychotic experiences are thought to lie on a continuum with psychotic disorders (9) and have been shown to predict elevated rates of schizophrenia by age 38 (10) and share many of the risk factors commonly associated with psychotic disorders (11). Moreover, these sub-clinical psychotic phenomena have also been shown to increase the risk for other psychiatric disorders (12), suicide attempts (10), and poor functional outcomes (13) in adulthood.

Therefore, one approach to informing early preventive interventions for psychotic disorders and potentially other adverse outcomes is to investigate factors that protect at-risk children and adolescents from developing psychotic experiences. A widely replicated risk factor for psychosis is having a family member with the disorder; for instance, having a parent with schizophrenia increases a child's risk of developing schizophrenia themselves by approximately eight-fold (14). A previous analysis of the Environmental Risk (E-Risk) Longitudinal Twin Study, a nationally-representative general population sample of twins, has shown that children were over twice as likely to report psychotic experiences at age 12 if their mother had experienced a psychosis-spectrum disorder (11). This indicates that young people whose mother has experienced psychosis could be considered a high-risk group for the development of early psychotic phenomena. However, not all offspring of affected mothers developed psychotic experiences (11) and thus it is important to understand what protected these high-risk individuals in order to inform preventive interventions.

In the E-Risk sample, it has previously been shown that, in the context of poly-victimization, having better cognitive functioning, growing up in a happier and more stimulating family environment, and living in a cohesive community, were protective against psychotic experiences among children (15), while engaging in physical activity, social support, and living in a cohesive community were protective for adolescents (16). However, it is also important to determine protective factors for other high-risk groups, such as having a parent with psychosis. Therefore, in the current study we use the E-Risk cohort to investigate whether these factors can also protect against the development of sub-clinical psychotic phenomena among children and adolescents who are at particularly high-risk of psychosis, by virtue of having a mother with a psychotic disorder or psychotic symptoms. We explored whether these factors were protective over and above potential confounding factors, including family socioeconomic status (SES) and other childhood mental health problems, which have previously been shown to be associated with the development of psychotic phenomena (11).

## Materials and Methods

### Participants

Participants were members of the E-Risk Longitudinal Twin Study, which tracks the development of a nationally-representative birth cohort of 2,232 British twin children. The sample was drawn from a larger cohort of twins born in England and Wales in 1994–1995 (17). Full details about the sample are recorded elsewhere (18). Briefly, the E-Risk sample was constructed in 1999–2000, when 1,116 families with same-sex 5-year-old twins (93% of those eligible) participated in home-visit assessments. Families were recruited to represent the UK population of families with new-borns in the 1990s, based on residential location throughout England and Wales and mothers' age. Teenaged mothers with twins were over-selected to replace high-risk families who were selectively lost to the register through non-response. Older mothers having twins via assisted reproduction were under-selected to avoid an excess of well-educated older mothers. E-Risk families are representative of UK households across the spectrum of neighborhood-level deprivation: 25.6% of E-Risk families live in “wealthy achiever” neighborhoods compared to 25.3% of households nation-wide; 5.3 vs. 11.6% live in “urban prosperity” neighborhoods; 29.6 vs. 26.9% live in “comfortably off” neighborhoods; 13.4 vs. 13.9% live in “moderate means” neighborhoods; and 26.1 vs. 20.7% live in “hard-pressed” neighborhoods (19, 20). E-Risk families under-represent “urban prosperity” neighborhoods because such households are likely to be childless. The sample comprised 56% monozygotic and 44% dizygotic twin pairs, and sex was evenly distributed within zygosity (49% male). All families were English speaking, and the majority (93.7%) were White.

Follow-up home-visits were conducted when children were aged 7, 10, 12, and 18 years (participation rates were 98, 96, 96, and 93%, respectively). Home visits at ages 5, 7, 10, and 12 years included assessments with participants as well as their mother (or primary caretaker); the home visit at age 18 included interviews only with the participants. Each twin participant was assessed by a different interviewer. The average age of the twins at the time of the age 18 assessment was 18.4 years (*SD* = 0.36); all interviews were conducted after the 18th birthday. There were no differences between those who did and did not take part at age 18 in terms of socioeconomic status (SES) assessed when the cohort was initially defined (χ^2^ = 0.86, *P* = 0.65), age-5 IQ scores (*t* = 0.98, *P* = 0.33), or age-5 internalizing or externalizing behavior problems (*t* = 0.40, *P* = 0.69 and *t* = 0.41, *P* = 0.68, respectively).

The Joint South London and Maudsley and the Institute of Psychiatry Research Ethics Committee approved each phase of the study. Parents gave informed consent and twins gave assent between 5 and 12 years and then informed consent at age 18.

### Measures

#### Maternal Psychosis-Spectrum Disorder

When children were aged 10, mothers were interviewed using the Diagnostic Interview Schedule (DIS) for DSM-IV (21), which enquires about characteristic symptoms of psychosis: hallucinations, delusions, disorganized speech, grossly disorganized, or catatonic behavior and negative symptoms (avolition, flat affect, alogia). They were asked whether they had experienced these symptoms at any time in their life. The interview ruled out symptoms with plausible explanations and symptoms occurring solely under the influence of alcohol or drugs. Following DSM-IV criteria for schizophrenia, women were classified as having a psychosis-spectrum disorder given the presence of hallucinations plus at least two other symptoms, as well as evidence of social, occupational, or self-care dysfunction (12). The goal was not to diagnose clinical schizophrenia, but to identify women who endorsed impairing psychotic-like experiences and beliefs. Of the 1,060 mothers who completed the DIS, 58 (5.5%) were classified as having a psychosis-spectrum disorder.

#### Maternal Psychotic Symptoms

When children were aged 12, mothers were interviewed with the Psychosis Screening Questionnaire (PSQ) (22) about psychotic symptoms that they experienced over the past 2 years. The PSQ consists of 6 main items covering symptoms of hypomania, thought insertion, paranoia, strange experiences and hallucinations, and 14 follow-up items. The six main items were all presented first and, if any main items were endorsed, the appropriate follow-up questions were asked. A symptom was considered present if the mother positively endorsed the main item and its follow-up questions. The number of psychotic symptoms was summed and then dichotomized into none vs. 1 or more psychotic symptoms due to the skewed nature of the variable. Of the 1,069 mothers who completed the PSQ, 177 (16.6%) reported one or more psychotic symptoms.

#### Childhood Psychotic Symptoms

E-Risk families were visited by mental health trainees or professionals when children were aged 12 (11). Each child was privately interviewed about 7 psychotic symptoms pertaining to delusions and hallucinations, with items including “have other people ever read your thoughts?,” “have you ever thought you were being followed or spied on?,” and “have you ever heard voices that other people cannot hear?.” This interview has been described in detail previously (11). The item choice was guided by the Dunedin Study's age-11 interview protocol (12) and an instrument prepared for the Avon Longitudinal Study of Parents and Children (23). Interviewers coded each experience 0, 1, 2 indicating, respectively “not a symptom,” “probable symptom,” and “definite symptom.” A conservative approach was taken in designating a child's report as a symptom. First, the interviewer probed using standard prompts designed to discriminate between experiences that were plausible (e.g., “I was followed by a man after school”) and potential symptoms (e.g., “I was followed by an angel who guards my spirit”) and wrote down the child's narrative description of the experience. Second, items and interviewer notes were assessed by a psychiatrist expert in schizophrenia, a psychologist expert in interviewing children, and a child and adolescent psychiatrist to verify the validity of the symptoms. Third, because children were twins, experiences limited to the twin experience (e.g., “My twin and I often know what each other are thinking”) were coded as “not a symptom.” Children were only designated as experiencing psychotic symptoms if they reported at least one definite psychotic symptom. At age 12, 5.9% (*N* = 125) of children reported experiencing psychotic symptoms. This is similar to the prevalence of psychotic symptoms in other community samples of children and adolescents (8, 24–27). Furthermore, our psychotic symptom measure has good construct validity, sharing many of the genetic, social, neurodevelopmental, and behavioral risk factors and correlates as adult schizophrenia (11).

#### Adolescent Psychotic Phenomena

The present study used two measures of adolescent psychotic phenomena which were both obtained from private interviews when participants were aged 18. The primary outcome was a self-report measure of adolescent psychotic *experiences* which reflects the methodology used by many groups in the psychosis prodromal research field (28). At age 18, each E-Risk participant was privately interviewed by a research worker about 13 psychotic experiences occurring since age 12. Seven items pertained to delusions and hallucinations, which were the same as those used in childhood (see above). This interview has been described in detail previously (11). Six items pertained to unusual experiences, which drew on item pools since formalized in prodromal psychosis instruments including the PRIME-screen and Structured Interview for Prodromal Syndromes (28). These included “I worry that my food may be poisoned” and “My thinking is unusual or frightening.” Interviewers coded each item 0, 1, 2 indicating, respectively “not present,” “probably present,” and “definitely present.” All 13 items were summed to create a psychotic experiences scale (range = 0–18, *M* = 1.19, SD = 2.58). Just over 30% of participants had at least one psychotic experience between ages 12 and 18 (*n* = 623, 30.2%). This is similar to the prevalence of self-reported psychotic experiences in other community samples of teenagers and young adults (8, 25).

Clinician-verified adolescent psychotic *symptoms* were also examined as a secondary outcome, using the same methodology as used at age 12 in this cohort (11). Adolescents were only designated as having psychotic *symptoms* if they reported at least one definite and verified symptom. At age 18, 2.9% (*N* = 59) of adolescents reported having one or more psychotic symptoms since age 12. This is somewhat lower than the prevalence of psychotic symptoms in this sample at age 12 (5.9%, *N* = 125), consistent with the attenuation of psychotic symptoms documented from childhood to adulthood (8, 29).

#### Childhood Protective Factors

##### Iq

The Wechsler Preschool and Primary Scale of Intelligence Revised (WPSSI; (30) was used to assess IQ at age 5. Children were administered two subtests (Vocabulary and Block Design), and IQ scores were prorated following procedures described previously (31) and then standardized with a mean of 100 and standard deviation of 15.

##### Atmosphere at home

The creation of the atmosphere at home measure has been previously documented (32). It was derived from the Coder's Impression Inventory, which is based on the Home Observation for Measurement of the Environment (33) and the University of Washington Parenting Clinic Questionnaire (Parent-Child Observations) (34). The Coder's Impression Inventory was rated immediately following the study visit at ages 7 and 10 by interviewers who had undergone 4-day training. This measure comprised items representing the state of the home (e.g., “Are visible rooms of the house clean?”), stimulation (e.g., “Is the children's art displayed in the home?”), happiness (e.g., “Is this a happy home?”), and chaos (e.g., “Is the house chaotic or overly noisy?”). The internal consistency at age 7 was α = 0.77 and α = 0.79 at age 10. The average of the overall atmosphere at home scores at ages 7 and 10 was used for analysis because they were significantly correlated (*r* = 0.64, *P* < 0.001).

##### Neighborhood social cohesion

Social cohesion within the neighborhood (35) was assessed when children were aged 5 by asking mothers five questions, including whether their neighborhood was close-knit, whether neighbors shared values, and whether neighbors trusted and got along with each other. A total score was derived by summing the answers to all five questions, with higher scores indicative of greater social cohesion.

#### Adolescent Protective Factors

##### Physical activity

At age 18, participants completed the Stanford Brief Activity Survey (SBAS; Stanford University, 2001). The SBAS contains 2 items, the first item relates to the extent of physical activity engaged in at work, school, or college and the second refers to physical activity during leisure time. Both questions were rated on a 5-point scale: inactive, low intensity, moderate intensity, hard intensity, and very hard intensity. The scales were then combined to derive an overall activity measure (36).

##### Neighborhood social cohesion

Social cohesion in the participants' neighborhoods was estimated via a postal survey sent to residents living alongside E-Risk families when participants were aged 13–14 (37, 38). Survey respondents, who were typically living on the same street or within the same apartment block as the participants in the study, reported on various characteristics of their immediate neighborhood. Five items (each coded 0–4) were assessed by asking residents whether their neighbors shared values and trusted and got along with each other, etc. A total score was derived by summing the answers to all 5 questions with higher scores indicating greater social cohesion.

##### Social support

Perceived social support was assessed at age 18 using the Multidimensional Scale of Perceived Social Support (MSPSS), which assesses individuals' access to supportive relationships with family, friends, and significant others (39). The 12 items in the MSPSS consist of statements such as “There is a special person who is around when I am in need” and “I can count on my friends when things go wrong.” Participants rated these statements as “not true” (0), “somewhat true” (1) or “very true” (2). Scores were summed to produce an overall social support scale with higher scores reflecting greater social support (internal consistency: α = 0.88).

#### Confounding Variables

Family SES was measured via a composite of parental income (total household), education (highest for mother/father), and occupation (highest for mother/father) when children were aged 5 (40). The three SES indicators were highly correlated (r's ranged from 0.57 to 0.68, all p's < 0.05) and loaded significantly onto one latent factor (factor loadings = 0.82, 0.70, and 0.83 for income, education, and occupation, respectively). This latent factor was categorized into tertiles (i.e., low-, medium-, and high-SES). A variable for childhood mental health problems was derived to capture children who met criteria for extreme anxiety, clinically-relevant depression symptoms, attention deficit hyperactivity disorder (ADHD), or conduct disorder by age 12. Anxiety was assessed when children were aged 12, via private interviews using the 10-item version of the Multidimensional Anxiety Scale for Children (MASC) (41). An extreme anxiety group was formed with children who scored at or above the 95th percentile (*N* = 129, 6.1%). Depression symptoms were assessed at age 12 using the Children's Depression Inventory (CDI) (42). Children who scored 20 or more were deemed to have clinically significant depressive symptoms (*N* = 74, 3.5%). ADHD was assessed using the DSM-IV and the requirement of symptom onset prior to age 12 was met if parents or teachers reported more than 2 ADHD symptoms at ages 5, 7, 10, or 12 years. We derived diagnoses of conduct disorder on the basis of mothers' and teachers' reports of children's behavior problems using the Achenbach family of instruments and additional DSM-IV items assessing conduct disorder which have previously been described (32). Conduct disorder was assumed present if it was diagnosed at ages 5, 7, 10, or 12 years. The childhood mental health problems variable was dichotomized to distinguish between the presence of any of the above mental health problems (coded 1) vs. the absence of any age-12 mental health problems (coded as 0), as used previously in this cohort (16).

### Statistical Analysis

Analyses were conducted in STATA 15 (Stata-Corp, College Station, TX). Bivariate and multivariate logistic regression analyses were conducted to explore associations between individual, family, and community-level putative protective factors and absence of age-12 and age-18 psychotic phenomena, in the E-Risk sub-sample of children whose mothers had experienced psychosis. Maternal psychosis was defined as either diagnosis of psychosis-spectrum disorder and/or presence of one or more psychotic symptoms to utilize the largest possible group of mothers experiencing psychotic phenomena (*N* = 408).

In phase 1 (age-12 analysis), binary logistic regression analyses were conducted to explore associations between each protective factor (IQ, atmosphere at home, and neighborhood social cohesion), individually and then altogether, and the absence of age-12 psychotic symptoms in the sub-sample of children whose mothers had experienced psychosis. We also tested in the whole sample for interactions between maternal psychosis status and each protective factor to examine whether these factors were specifically protective in relation to having a mother who had experienced psychotic phenomena or were more generally protective.

In phase 2 (age-18 analysis), binary logistic regression analyses were conducted to explore associations between each protective factor (physical activity, neighborhood social cohesion, and perceived social support), individually and then altogether, and the absence of age-18 psychotic experiences in the sub-sample of children whose mothers had experienced psychosis. Interactions between maternal psychosis status and each protective factor were conducted to examine whether these factors were specifically protective in relation to having a mother who had experienced psychotic phenomena. Sensitivity analyses substituting age-18 psychotic experiences with the clinician-verified psychotic symptoms at age 18 were also conducted.

Because each study family contains two children, all statistical analyses were corrected conservatively for the non-independence of twin observations by using tests based on the Huber/White variance estimator (43). All analyses were also adjusted for child's gender and family socio-economic status because these factors have previously been associated with psychosis (44, 45). Age-18 analyses were also adjusted for age-12 psychotic symptoms and childhood mental health problems as these have been shown to predict the occurrence of psychotic phenomena in adolescence (29, 46).

## Results

### Phase 1: Age-12

#### Sample

The sample (*N* = 2,182) was 51.1% (*N* = 1,114) female. Family SES was categorized into low- (33.2%, *N* = 724), medium- (32.8%, *N* = 716), and high-SES (34.0%, *N* = 742). Having a mother with either a diagnosis of psychosis-spectrum disorder or at least one psychotic symptom was associated with an increased likelihood of age-12 psychotic symptoms among their children (OR = 2.14, 95% CI 1.38–3.31, *P* = 0.001).

#### Associations Between Putative Protective Factors and Absence of Age-12 Psychotic Symptoms Among Mothers With Psychosis

Having a higher IQ, a more positive atmosphere in the home, and residing in a more socially cohesive neighborhood were all found to be significantly protective against age-12 psychotic symptoms among children of mothers who had experienced either psychotic symptoms or had a psychosis-spectrum diagnosis after adjusting for gender and family SES (Table 1). When additionally controlling for the other protective factors, higher IQ (OR = 0.97, 95% CI 0.94–1.00, *P* = 0.036) and neighborhood social cohesion (OR = 0.88, 95% CI 0.79–0.98, *P* = 0.023) remained significantly associated with a reduced likelihood of the child having psychotic symptoms, but the association for atmosphere at home became non-significant (OR = 0.96, 95% CI 0.92–1.01, *P* = 0.133).

**Table 1:** Associations between putative protective factors and absence of age-12 psychotic symptoms among children of mothers with diagnosis or symptoms of psychosis.

**Putative protective factor**	**Psychotic symptoms absent *N* = 359**	**Psychotic symptoms present *N* = 40**	**Adjusted OR^a^**	**95% CI**	***P***
	***M (SD)***	***M (SD)***			
IQ	97.84 (14.77)	88.88 (14.58)	0.96	0.94–0.99	0.007
Atmosphere at home	23.32 (6.92)	19.51 (6.96)	0.93	0.89–0.98	0.003
Neighborhood social cohesion	7.14 (3.02)	5.30 (3.28)	0.85	0.76–0.96	0.007

*CI, confidence interval; IQ, intelligent quotient; OR, odds ratio; SD, standard deviation*.

a *Adjusted for gender and family socioeconomic status. All analyses account for the non-independence of twin observations*.

#### Interactions Between Maternal Psychosis Status and Protective Factors in the Whole Sample

Mean IQ (*M* = 101.1, SD = 14.8), atmosphere at home (*M* = 26.6, SD = 4.8) and neighborhood social cohesion (*M* = 7.8, SD = 2.6) were highest when neither the child nor mother had symptoms or diagnosis of psychosis. Mean scores were lowest when both mother and child had psychotic symptoms or diagnosis: IQ (*M* = 88.9, SD = 14.6); atmosphere at home (*M* = 19.5, SD = 7.0); neighborhood social cohesion (*M* = 5.3, SD = 3.3). However, none of the interactions between maternal psychosis status and the protective factors were statistically significant (Table 2). Indeed, higher average IQ (OR = 0.98, 95% CI 0.96–0.99, *P* = 0.002) and a more positive atmosphere at home (OR = 0.93 95% CI 0.89–0.97, *P* = 0.002) were significantly associated with a reduced likelihood of age-12 psychotic symptoms in children of mothers *without* psychosis, with a non-significant trend for greater neighborhood social cohesion (OR = 0.93, 95% CI 0.85–1.01, *P* = 0.071).

**Table 2:** Interactions between maternal psychosis and putative protective factors in relation to the absence of age-12 psychotic symptoms and age-18 psychotic experiences in the whole sample.

**Interaction with maternal psychosis**	**Adjusted OR**	**95% CI**	***P***
**AGE-12**			
IQ	0.98	0.95–1.01	0.300
Atmosphere at home	1.01	0.96–1.07	0.739
Neighborhood social cohesion	0.92	0.81–1.06	0.247
**AGE-18**			
Physical activity	1.08	0.87–1.34	0.494
Neighborhood social cohesion	0.86	0.51–1.43	0.553
Social support	1.01	0.94–1.07	0.818

*CI, confidence interval; IQ, intelligent quotient; OR, odds ratio. All analyses are adjusted for gender and family socioeconomic status; age-18 analyses are additionally adjusted for age-12 psychotic symptoms and other age-12 mental health problems. All analyses account for the non-independence of twin observations*.

### Phase 2: Age-18

#### Sample

The sample (*N* = 2,041) was 52.3% (*N* = 1,067) female. Family SES was categorized into low- (33.4%, *N* = 681), medium- (33.1%, *N* = 675), and high-SES (33.5%, *N* = 685). Having a mother with either a diagnosis of psychosis-spectrum disorder or at least one psychotic symptom was associated with an increased likelihood of age-18 psychotic experiences among their children (OR = 1.56, 95% CI 1.21–2.01, *P* = 0.001).

#### Are Protective Factors Associated With an Absence of Age-18 Psychotic Phenomena Among Adolescents Whose Mother has Psychosis?

Greater levels of perceived social support were significantly associated with a reduced likelihood of adolescent psychotic experiences when controlling for all confounders among children of mothers with a psychosis-spectrum disorder or psychotic symptoms (Table 3). No associations were evident for physical activity, but there was a non-significant trend for greater neighborhood social cohesion to be associated with a reduced likelihood of adolescent psychotic experiences (OR = 0.67, 95% CI 0.42–1.08, *P* = 0.101). When additionally controlling for the other protective factors, higher levels of perceived social support remained significantly associated with a reduced likelihood of adolescents having psychotic experiences (OR = 0.92, 95% CI 0.87–0.98, *P* = 0.006).

**Table 3:** Associations between putative protective factors and absence of age-18 psychotic experiences among children of mothers with a diagnosis or symptoms of psychosis.

**Putative protective factor**	**Psychotic experiences absent *N* = 238**	**Psychotic experiences present *N* = 144**	**Adjusted OR^a^**	**95% CI**	***P***
	***M (SD)***	***M (SD)***			
Physical activity	2.71 (1.09)	2.72 (1.09)	1.02	0.83–1.25	0.871
Neighborhood social cohesion	2.24 (0.52)	2.09 (0.50)	0.67	0.42–1.08	0.101
Social support	21.02 (4.55)	18.63 (4.99)	0.92	0.87–0.98	0.006

*CI, confidence interval; OR, odds ratio; SD, standard deviation*.

a *Adjusted for gender, family socioeconomic status, age-12 psychotic symptoms, and other age-12 mental health problems. All analyses account for the non-independence of twin observations*.

Sensitivity analyses were then conducted substituting the psychotic experiences outcome with the rarer clinician-verified psychotic symptoms at age 18. None of the protective factors were found to be significantly associated with a reduced likelihood of psychotic symptoms being reported at age 18 when controlling for potential confounders among the adolescents whose mothers had psychosis (Table 4). However, the effect sizes were similar to those for psychotic experiences, and there was a non-significant trend for greater neighborhood social cohesion to be associated with a reduced likelihood of adolescent psychotic symptoms (OR = 0.62, 95% CI 0.22–1.69, *P* = 0.346).

**Table 4:** Associations between putative protective factors and an absence of age-18 psychotic symptoms among adolescents whose mothers had a psychosis-spectrum disorder or psychotic symptoms.

**Putative protective factor**	**Psychotic symptoms absent *N* = 360**	**Psychotic symptoms present *N* = 22**	**Adjusted OR^**a**^**	**95% CI**	***P***
	***M (SD)***	***M (SD)***			
Physical activity	2.71 (1.08)	2.68 (1.25)	1.00	0.64–1.58	0.991
Neighborhood social cohesion	2.19 (0.50)	2.00 (0.68)	0.62	0.22–1.69	0.346
Social support	20.16 (4.88)	19.36 (4.38)	0.99	0.93–1.07	0.882

*CI, confidence interval; M, mean; OR, odds ratio; SD, standard deviation*.

a *Adjusted for gender, family socioeconomic status, age-12 psychotic symptoms, and other age-12 mental health problems. All analyses account for the non-independence of twin observations*.

#### Are Protective Factors Only Associated With an Absence of Age-18 Psychotic Experiences Among Adolescents Whose Mother has Psychosis?

There were no significant interactions between each of the protective factors and maternal psychosis status in relation to an absence of age-18 psychotic experiences in the full sample (Table 2). Indeed, higher levels of perceived social support were also associated with a reduced likelihood of age-18 psychotic experiences in children of mothers *without* psychosis (OR = 0.91, 95% CI 0.89–0.94, *P* < 0.001).

## Discussion

### Summary of Findings

Higher IQ and greater neighborhood social cohesion were found to be independently protective against the development of age-12 psychotic symptoms among children of mothers with a psychosis-spectrum disorder or psychotic symptoms. At age 18, higher levels of perceived social support were found to be independently protective of psychotic experiences among adolescents of mothers with a psychosis-spectrum disorder or psychotic symptoms. There was a non-significant trend for neighborhood social cohesion to be protective for adolescent psychotic experiences. As none of the interactions between protective factors and maternal psychosis status investigated were found to be significant, this indicates that factors were not specifically protective against psychotic phenomena amongst these high-risk children. Indeed, all of the factors were also found to be protective amongst children and adolescents whose mothers had not experienced psychotic phenomena.

### Comparison to Previous Research

Our results are consistent with previous research on the E-Risk study which reported that higher IQ, a more positive atmosphere at home, and greater neighborhood social cohesion were associated with a reduced likelihood of childhood psychotic symptoms in the whole sample and not just in high-risk children exposed to multiple forms of victimization (15). Similarly, previous studies show that possessing a higher IQ is associated with a reduced likelihood of developing psychotic symptoms (47, 48); while lower childhood IQ is associated with a range of mental health problems, including an increased risk of developing schizophrenia, depression and anxiety as an adult (49). Our findings also suggest that high levels of neighborhood social cohesion are independently protective in children with mothers who have either symptoms or a diagnosis of psychosis, and in the whole population. This result is consistent with previous research showing that low levels of social cohesion are associated with greater odds of developing psychotic symptoms (50–52). Additionally, our findings highlight the protective effect of perceived social support in adolescence which is consistent with previous research in E-Risk showing that social support was associated with a reduced likelihood of psychotic experiences among high-risk adolescents who had been poly-victimized (16). A study of clinical-level psychosis also demonstrated the protective nature of social support among high-risk individuals (53).

### Strengths and Limitations

Strengths of the study include our novel analysis that examines protective factors for children and adolescents who are at high risk of psychosis by having a mother with a psychotic disorder or psychotic symptoms. Furthermore, our participants were taken from a large, nationally-representative longitudinal cohort study that measured psychotic phenomena at two time-points and enabled us to control for a range of potentially confounding factors. Several limitations warrant consideration. The sample size was relatively small when focusing on children of mothers with psychosis and so we may have lacked statistical power to detect significant effects, especially for the interaction analysis. Moreover, it was not possible to explore protective effects in children who mothers had a diagnosis of psychosis-spectrum disorder separately to those whose mothers had psychotic symptoms due to the small number of children in the former group (*N* = 58). The sample was composed of twins and so we cannot be certain if the findings can be generalized to single children, although the prevalence of childhood and adolescent psychotic phenomena in this study are similar to rates found in singleton samples (8, 25, 27). We limited our analyses to factors that have previously been found to be protective for psychotic phenomena in this cohort and it is possible that a range of other multi-level factors may also be protective. For instance, self-esteem (54), attachment style (55), and positive behavioral support (56). These require further investigation ideally in even larger population-based cohorts. In adolescence several of the measures were assessed at the same time-point and therefore it is not possible to ascertain the direction of the associations found. We did however control for earlier psychopathology, but it would be useful in future to utilize prospective measures, ideally obtained from different informants. Additionally, psychotic phenomena in childhood and adolescence have been associated not only with later development of schizophrenia but also with other mental health problems (10, 12), and thus the findings cannot specifically be generalized to indicate protection against clinically-relevant psychosis. Finally, our analyses focused on psychotic phenomena assessed only at the ages of 12 and 18 so we do not know if the factors analyzed would be protective if symptoms were to develop at other ages in childhood or adolescence, or in adulthood.

### Clinical Applications and Future Research

The findings of this study have the potential to inform the focus of interventions to prevent the emergence of early psychotic phenomena and thus ultimately improve the outcomes of high-risk children and adolescents. Intellectual ability, neighborhood social cohesion, and perceptions of social support appear to be key target areas for early intervention. It is possible that possessing a higher IQ may facilitate problem-solving skills, coping strategies in adverse situations, and better self-regulation of emotions, particularly in relation to having a parent with psychosis. Cognitive behavioral therapies are being developed for young people that aim to target and develop skills such as reasoning and emotional coping, as a way of increasing resilience (57). Cognitive remediation therapy is another approach that may offer a way to alleviate cognitive difficulties in young people (58). Additionally, neighborhood support interventions to improve parenting have been demonstrated in a study which found high levels of neighborhood social cohesion were associated with reduced instances of child neglect (59). Such an outcome could occur through alleviating the burden that comes with childcare for mothers with psychosis as neighbors meet the physical and emotional needs of both child and parent. Trust between neighbors may also mean that children approach them for help when they are feeling distressed, providing further adaptive ways of coping (60). Research suggests that when people with early psychosis perceive greater social support, they appear to be more likely to cope with day-to-day stressors (61). Higher levels of social support have been found to correlate with lower levels of positive symptoms and fewer hospitalizations in people with first episode psychosis (62). Therefore, if the current findings are replicated in other cohorts, it would be helpful to investigate whether such interventions might prevent the development of psychotic phenomena in children and adolescents.

## Conclusions

Higher IQ and neighborhood social cohesion were found to be independently associated with a reduced likelihood of age-12 psychotic symptoms among children of mothers with symptoms of psychosis or a psychosis-spectrum diagnosis. Similarly, higher levels of perceived social support were found to be independently protective against adolescent psychotic experiences in this high-risk group. In terms of clinical implications, if replicated, the findings suggest that interventions should be aimed at cultivating these factors for children and adolescents who are considered at-risk by virtue of having a mother with psychosis. All of these factors were also found to be associated with a reduced likelihood of having psychotic phenomena among children whose mother had not experienced psychosis, which suggests that preventative interventions could also be targeted at the general population regardless of risk. These preventative interventions should improve the cohesiveness of a child's community environment, increase their perceptions of social support, and bolster their cognitive functioning. Replication of these findings is required in larger populations, amongst single children, in order to ascertain generalizability and subsequently inform the development and testing of interventions to prevent the emergence of early psychotic phenomena. Ultimately, such preventive strategies may reduce the incidence of psychosis and other mental health problems in adulthood.

## Author Contributions

HF designed the project and oversaw the analyses and write-up of the manuscript. RB-N, MA, and SR conducted the analyses and wrote the first draft of the manuscript. LA oversaw data collection for the E-Risk Study. All authors critically reviewed and amended the content of the manuscript and approved the final version.

### Conflict of Interest Statement

The authors declare that the research was conducted in the absence of any commercial or financial relationships that could be construed as a potential conflict of interest.
